# Massive Haemoptysis due to Obscure Aetiology: Perils and Management Dilemmas

**DOI:** 10.1155/2018/8159896

**Published:** 2018-11-27

**Authors:** A. Shreenivasa, K. A. Vishak, K. Sindhu, Kauslya Sahu, G. V. Chaithra

**Affiliations:** ^1^Senior Resident, Department of Pulmonary Medicine, KMC Mangalore, MAHE University, Udupi, India; ^2^Professor, Department of Pulmonary Medicine, KMC Mangalore, MAHE University, Udupi, India; ^3^Professor, Department of Pathology, KMC Mangalore, MAHE University, Udupi, India; ^4^Assistant Professor, Department of Pathology, KMC Mangalore, MAHE University, Udupi, India

## Abstract

Pulmonary actinomycosis is an important differential diagnosis in patients with long-standing pulmonary infiltrates related to poor oral hygiene or compromised immune function. Up to a quarter of cases of thoracic actinomycosis are misdiagnosed as lung malignancy. Here, we report a 56-year-old man with a hypodense lesion in the left lower lobe presenting with recurrent massive haemoptysis for about one year. He underwent left lower lobe lobectomy due to intractable haemoptysis. Histopathological examination demonstrated actinomycosis infiltrating the left lower lobe. Rarity of the case was the presence of actinomycosis in an immunocompetent individual and without underlying preexisting lung disease. Also, intractable massive haemoptysis necessitating surgical excision which proved to be both diagnostic and curative due to actinomycosis is an unusual occurrence.

## 1. Background

Actinomycosis is an uncommon, chronic, and slowly progressive bacterial infection caused by *Gram-positive anaerobic bacteria*, belonging to the family Actinomycetaceae [1]. In humans, actinomycosis is associated with suppurative and granulomatous inflammation characterized by swelling, sinus tract formation, and purulent discharge containing yellowish sulfur granules [2]. Pulmonary actinomycosis accounts for 15–20% total incidence of actinomycosis [3]. Actinomyces are thought to colonize devitalized tissues; thus, higher incidence of pulmonary actinomycosis has been reported in patients with underlying lung disorders, such as emphysema, chronic bronchitis, and bronchiectasis [4].

Pulmonary actinomycosis is caused by aspiration of oropharyngeal or gastrointestinal secretions into the respiratory tract. Pulmonary actinomycosis usually presents with chest pain, productive cough, and dyspnea [5]. Pulmonary actinomycosis is rarely included in the differential diagnosis of a patient with pulmonary infiltrates [6], with most patients being investigated for other possible diseases, before the final diagnosis is made.

## 2. Case Report

A 53-year-old male, smoker (20 pack-years), occasional alcoholic, presented with complaints of blood expectoration of 400 ml in one episode followed by 100–150 ml for 2–3 days. He had 3 episodes of similar history which required hospitalizations and emergency care since 9 months. He denied history of fever, chest pain, and loss of appetite. He underwent cholecystectomy 3 years ago. There was no history of systemic immune suppression like diabetes. He had undergone bronchial artery embolization for massive haemoptysis; however, his haemoptysis persisted and diagnosis remained elusive after evaluation with sputum studies and CT-guided aspiration cytology, biopsy, and bronchoscopic lavage. He was treated for LRTI with multiple courses of antibiotics for more than 9 months.

**Figure 1:**
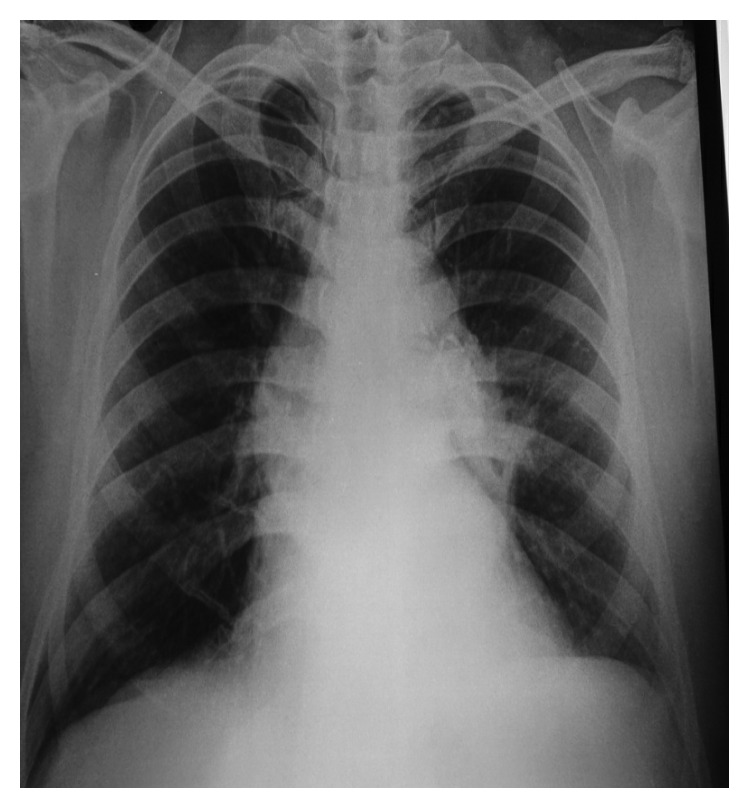
Chest X-ray revealed raised left hemidiaphragm and left midzone opacity, creating a positive silhouette sign with the left cardiac border.

**Figure 2:**
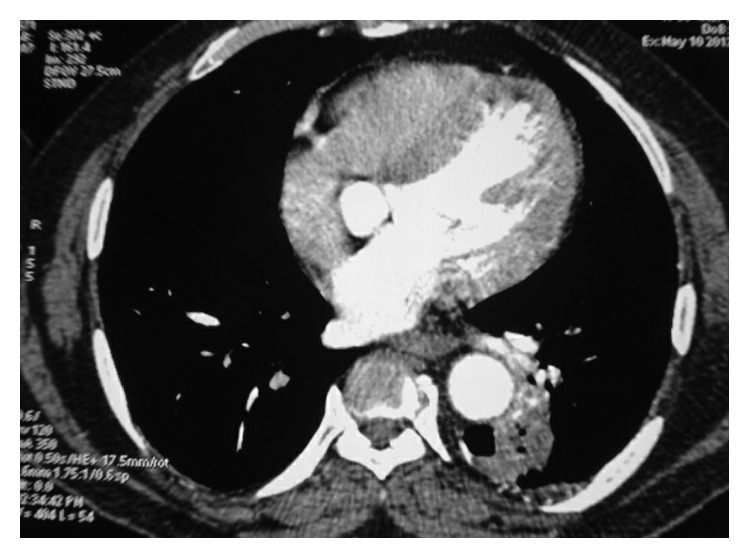
Irregular margin air space consolidation with cavitations seen in the left anterior segment adjacent to descending aorta.

**Figure 3:**
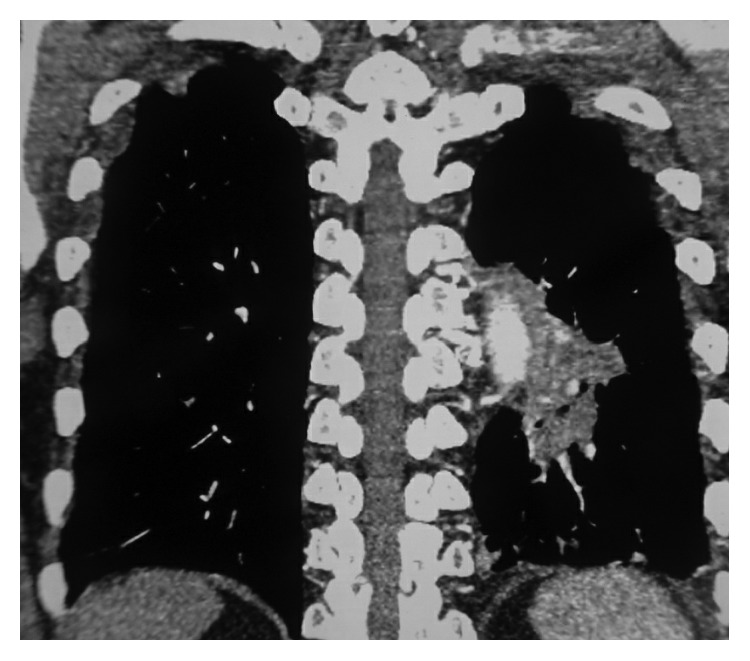
An irregular mass is seen on the left lower lobe adjacent to aorta with pleural involvement.

On examination, vitals were normal with no respiratory distress. Oral hygiene was poor with dental caries. Respiratory examination revealed scattered crackles in the left lower lobe area. Chest X-ray showed an inhomogeneous opacity in the left lower zone with raised left diaphragm (Figure 1), and CECT (contrast-enhanced computer tomogram) chest showed a hypodense lesion with irregular margins in the anterior segment of the left lower lobe adjacent to the descending aorta and associated subcarinal lymphadenopathy (Figures 2 and 3). Image-guided transthoracic biopsy showed type 2 alveolar cell hyperplasia with negative immunohistochemistry. Bronchoscopy confirmed left lower lobe bleed with any endoluminal lesion. Bronchial wash was negative for microbiological and cytological studies including AFB stain, geneXpert for MTB complex, and pyogenic culture. Patient's symptoms of haemoptysis persisted; hence, CT angiogram was performed, which showed dilated vascular channels within the lesion without any obvious extravasations of contrast and no aortic abnormality.

**Figure 4:**
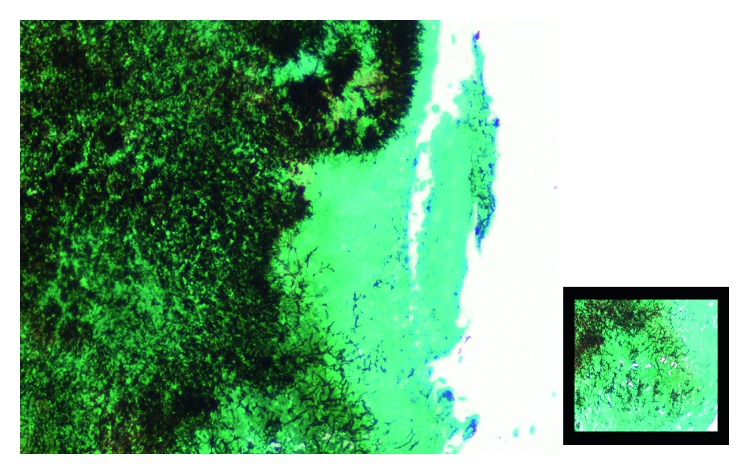
GMS stain highlighting delicate branched filaments.

Probable diagnosis of the left intrapulmonary vascular lesion was made and hence the patient underwent left lower lobe lobectomy. Intraoperatively, the left lower lobe was adherent posterolaterally to the aorta and diaphragm. Multiple prominent blood vessels in areas of adhesion were seen. Histopathology was suggestive of chronic inflammatory cells with focal aggregates of lymphocytes with positive GMS staining for actinomycosis (Figure 4). Postoperatively, the patient received parenteral benzylpenicillin 20 lakh units 6^th^ hourly for 3 months. The patient was in regular follow-up, and no further episodes of haemoptysis and no recent respiratory complaints are reported.

## 3. Discussion

Pulmonary actinomycosis is seen at all ages, commonly in adults and peak incidence described in the 4^th^ and 5^th^ decades [7]. In recent years, with the improvement in oral hygiene and early use of antibiotics, the presentation of pulmonary actinomycosis has changed from aggressive to less aggressive, making diagnosis more difficult [8]. Clinical manifestations of pulmonary actinomycosis are variable although cough and sputum are the most common symptoms [9]. Haemoptysis, although not common, has been reported often in pulmonary actinomycosis [2] and may be explained by the underlying structural diseases such as bronchiectasis. The disease usually affects the lower lobe [10], probably reflecting the role of aspiration in its pathogenesis. Pulmonary actinomycosis is usually characterized by a fibrotic lesion that is slowly progressive through the anatomical barriers which is often confused with malignancy [1].

Pulmonary actinomycosis shares many similar clinical features with chronic suppurative lung infections such as tuberculosis, fungal infections, and lung abscesses and also lung malignancy with which it is frequently confused [4]. Culture of bacteria from the sputum or bronchoalveolar secretions is technically difficult [11], and sometimes it also represents colonization of nonpathological microorganisms [12]. To confirm the diagnosis of pulmonary actinomycosis, lung biopsy is usually required [10].

Pulmonary actinomycosis may present as masses, nodules, patchy infiltrates, and solitary lesions [8]. A CT chest or ultrasound-guided biopsy is usually recommended prior to the surgical biopsy. However, the CT-guided biopsies may not be diagnostic as reported in our case. There could also be a case for percutaneous aspirated abscess to be sent for cultures routinely along with cytology to improve diagnostic accuracy. Thus, the gold standard for diagnosis of thoracic actinomycosis is a histological confirmation on lung biopsy.

Most cases of pulmonary actinomycosis have been diagnosed from postsurgical specimens taken on suspicion of lung cancer [1] as done in our case. Inadvertent use of antibiotics and nonspecific clinical presentations make pulmonary actinomycosis difficult to be diagnosed and often lead to misdiagnosis as malignancy rather than an infective disease [13]. Coexistence of lung cancer with pulmonary actinomycosis results in diagnostic challenge [14]. Reduction of alcohol abuse and improvement of dental hygiene may limit the occurrence of pulmonary actinomycosis [1] and require prolonged high-dose antimicrobial therapy with beta-lactam antibiotics for about 6–12 months [1].

In conclusion, this case is remarkable not only for the development of pulmonary actinomycosis in an immunocompetent patient but also for associated recurrent massive haemoptysis which eluded diagnosis and finally resection surgery confirmed the diagnosis and was also curative.

## References

[1] Valour F., Sénéchal A., Dupieux C. (2014). Actinomycosis: etiology, clinical features, diagnosis, treatment and management. *Infection and Drug Resistance*.

[2] Kim S. R., Jung L. Y., Oh I. J. (2013). Pulmonary actinomycosis during the first decade of 21st century: cases of 94 patients. *BMC Infectious Diseases*.

[3] Wong V. K., Turmezei T. D., Weston V. C. (2011). Actinomycosis. *BMJ*.

[4] Mabeza G. F., Macfarlane J. (2003). Pulmonary actinomycosis. *European Respiratory Journal*.

[5] Kim T. S., Han J., Koh W. J. (2006). Thoracic actinomycosis: CT features with histopathologic correlation. *American Journal of Roentgenology*.

[6] Alfaro T. M., Bernardo J., Garcia H. (2011). Organizing pneumonia due to actinomycosis: an undescribed association. *Respiration*.

[7] Heffner J. E. (1988). Pleuropulmonary manifestations of actinomycosis and nocardiosis. *Seminars in Respiratory Infections*.

[8] Sun X.-F., Wang P., Liu H.-R., Shi Ju-H. (2015). A retrospective study of pulmonary actinomycosis in a single institution in China. *Chinese Medical Journal*.

[9] Kwong J. S., Muller N. L., Godwin J. D., Aberle D., Grymaloski M. R. (1992). Thoracic actinomycosis: CT findings in eight patients. *Radiology*.

[10] Ahmed F., Teoh R., Kastelik J., Campbell A., McGivern D. (2009). Case series of thoracic actinomycosis presenting as a diagnostic challenge. *Respiratory Medicine CME*.

[11] Wong R. H., Sihoe A. D., Thung K. H., Wan I. Y., Ip M. B., Yim A. P. (2004). Actinomycosis: an often forgotten diagnosis. *Asian Cardiovascular and Thoracic Annals*.

[12] Ariel I., Breuer R., Kamal N. S., Ben-Dov I., Mogel P. (1991). RosenmannE Endobronchialactinomycosis simulating bronchogenic carcinoma. Diagnosis by bronchial biopsy. *Chest*.

[13] Qiu L., Lan L., Feng Y., Huang Z., Chen Y. (2015). Pulmonary actinomycosis imitating lung cancer on ^18^F-FDG PET/CT: a case report and literature review. *Korean Journal of Radiology*.

[14] Charif F., Harb A., Alifano M. (2009). Bronchial carcinoma and actinomycosis: a dangerous trap. *Revue des Maladies Respiratoires*.

